# MS-RIDD paves the way toward routine double bond localization in mass spectrometry-based lipidomics

**DOI:** 10.1038/s42004-022-00805-1

**Published:** 2022-12-29

**Authors:** Leonida Marion Lamp, Jürgen Hartler

**Affiliations:** 1 Institute of Pharmaceutical Sciences, University of Graz, Graz, Austria; 2 Field of Excellence BioHealth, University of Graz, Graz, Austria

**Keywords:** Lipidomics, Cheminformatics, Lipidomics, Mass spectrometry

## Abstract

Pinpointing double bond (C=C) positions in native lipid extracts is beyond the capabilities of standard mass spectrometry-based approaches. This article highlights a novel untargeted workflow supported by the open-source software MS-RIDD, that allows for semi-automated annotation of C=C locations with high confidence.

Lipids are a highly diverse group of compounds, both structurally and in terms of their functions in cells, tissues, and organs^1^. A substantial portion of the lipidome is composed of unsaturated lipids, i.e., lipids with at least one double bond (C=C) in one or more of their constituent hydrophobic lipid chains (this includes fatty acyl and ether chains as well as sphingoid bases). Consequently, isomers resulting from different C=C positions contribute significantly to the structural diversity of lipids^2^. The importance of specific lipid C=C positions in health and disease is well known, even in popular science, particularly with respect to omega-3 (n-3) and omega-6 (n-6) fatty acids^3^ (for omega-nomenclature, see Fig. 1a). However, most of the physiological and pathophysiological consequences of C=C isomerism remain concealed, as resolving C=C positions is not routine in lipidomics.

**Fig. 1:**
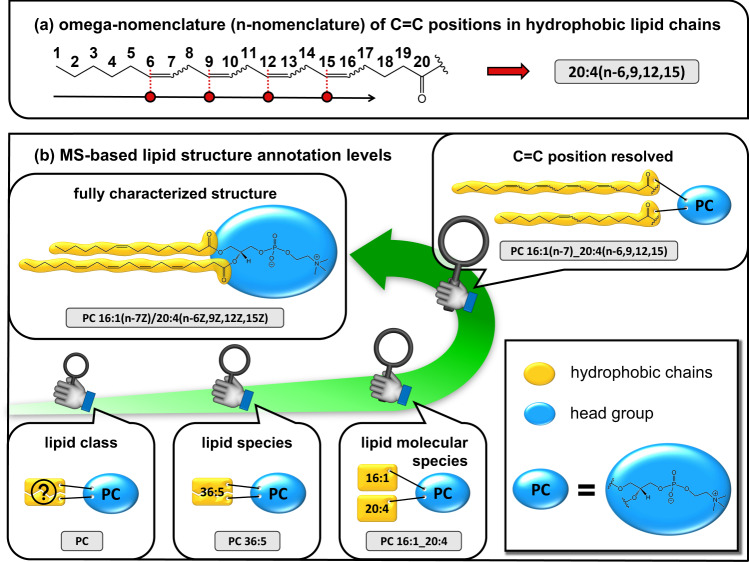
Lipid structure annotation levels including an illustration of the omega description. **a** omega-nomenclature illustrated by the example of arachidonic acid. According to the prevalent mass spectrometry (MS) shorthand nomenclature by Liebisch et al.^13^, arachidonic acid is referred to as 20:4 because it consists of 20 carbon atoms and four double bonds (C=C). In this notation, the n-nomenclature is indicated by brackets whose content starts with ‘n-‘ followed by the corresponding C=C positions. The numbering of the carbons starts at the methyl group. **b** MS-based structural annotation levels. Hydrophobic chains are indicated in gold, and blue ovals represent phosphocholine including the glycerol backbone. The lowest structurally resolved lipid identification is the lipid class level, which provides an aggregate measure for all species pertaining to a particular lipid class, e.g., in this case, phosphatidylcholine (PC). At this level, no information on the attached hydrophobic chains is available. The lipid species level resolves the constituent species of a class by their respective molecular mass. As for the structure, only the sum of the number of carbon atoms and the sum of C=C contained in the chains can be resolved. Structural elucidation at the lipid molecular species level involves knowledge of the chain length and number of C=C in each of the constituent hydrophobic lipid chains. The C=C position level resolves C=C locations. This information can be provided by the n-nomenclature or the delta-nomenclature (numbering starts at the terminal carbon atom, which is involved in the heteroatomic bond with the rest of the lipid molecule). Ultimately, complete structural characterization is achieved when positions on the glycerol backbone (*sn*-positions) and *cis–trans* isomerism are resolved.

Lipidomics has expanded rapidly since its beginnings in the early 2000s, driven by technological advances particularly in liquid chromatography (LC), mass spectrometry (MS), and computational methods^4^. However, structural elucidation of lipids by MS-based fragmentation spectra beyond the lipid molecular species level (Fig. 1b) remains a major challenge^5^. Commonly employed collision-induced fragmentation (CID) techniques are not suitable for C=C annotation, since they do not favor cleavages at C=C. Consequently, sophisticated fragmentation techniques have been developed to produce C=C position-specific ions (reviewed in Zhang et al.^5^). Despite these intriguing analytical advances, C=C position annotation is not commonly established in the community. A major obstacle seems to be the lack of automated computational tools since specifically, C=C fragmentation techniques produce highly complex spectra, which are particularly hard to deconvolute.

To provide computational means for C=C position annotation, Arita et al.^6^ developed the tool named mass spectrometry radical-induced dissociation decipherer (MS-RIDD), an open-source software tailored to a promising untargeted analysis strategy for complex biological samples. MS-RIDD owes its name to the fragmentation technique it was devised for: oxygen attachment dissociation (OAD)^7^, which belongs to the group of radical-induced fragmentation methods. For OAD, a radical source generates atomic oxygen (O) and hydroxyl radicals (OH), which are transferred to the collision cell of the mass spectrometry instrument. There, these radicals produce fragments specific for C=C positions. OAD offers two main advantages compared to alternative techniques. Firstly, it allows for the detection of lipids in their native form without requiring prior derivatization of analytes unlike e.g. methods based on the Paternò-Büchi reaction^8^. Secondly, OAD relies on water as a safe source for the radicals required to induce C=C cleavage, in contrast to hazardous ozone as used for ozonolysis^9^. However, OAD rarely reveals ions characteristic of intact hydrophobic lipid chains, which are required for lipid molecular species identification. To overcome this drawback, Arita and colleagues^6^ deftly devised a strategy that incorporates both CID spectra (in both polarities) and OAD spectra (positive ion mode) in one analytical workflow. The CID-related measurements reveal information up to the lipid molecular species level, which is further complemented by C = C position information obtained by OAD spectra.

To deal with the two types of MS data, Arita et al.^6^ integrated two computational tools in the analytical workflow (Fig. 2). For the processing of CID data, MS-DIAL^10^ is used, which works seamlessly with MS-RIDD for OAD spectra annotation. In the first step, all raw data are processed by MS-DIAL. This task involves peak picking, assignment of tandem mass spectrometry (MS/MS) spectra (fragmentation spectra) to the respective MS^1^ peaks as well as peak alignment. Notably, MS-DIAL processes the MS^1^ signals of both LC-CID-MS/MS and LC-OAD-MS/MS data to obtain information about intact lipid species. For CID data, MS-DIAL additionally annotates lipids up to the lipid molecular species level based on MS/MS spectra. At this stage, manual curation of these intermediate results is recommended. Subsequent to MS-DIAL processing, MS-RIDD receives a list of lipid molecular species from MS-DIAL to query the OAD data for C=C position information. To do that, MS-RIDD searches for fragment ion pairs diagnostic for C=C positions in the OAD MS/MS spectra. These obtained position detections are directly integrated into MS-DIAL’s lipid molecular species identifications, resulting in annotations at the C=C position resolved level as exemplified in Fig. 1b. Importantly, detected C=C positions can be unambiguously assigned to the respective chain in most cases since the C=C positions are characterized by neutral losses from the omega end, where each preceding C=C reduces the mass difference by 2 Da. Accordingly, the exact number of preceding C=C is known, which allows for the annotation of most lipid species consisting of more than one unsaturated chain. In this context, Arita and colleagues^6^ advise caution, as ambiguities may arise in the presence of cyclopropane-containing hydrocarbon chains since their OAD fragments are similar to those specific for C=C. Cases, where experimentally observed fragments result in ambiguous assignments, can be manually resolved on the basis of biological knowledge via a simple-to-use graphical user interface. In the present study, the authors benchmarked MS-RIDD on more than 20 lipid subclasses pertaining to the LIPID MAPS categories^11^ glycero-, glycerophospho- and sphingolipids.

**Fig. 2:**
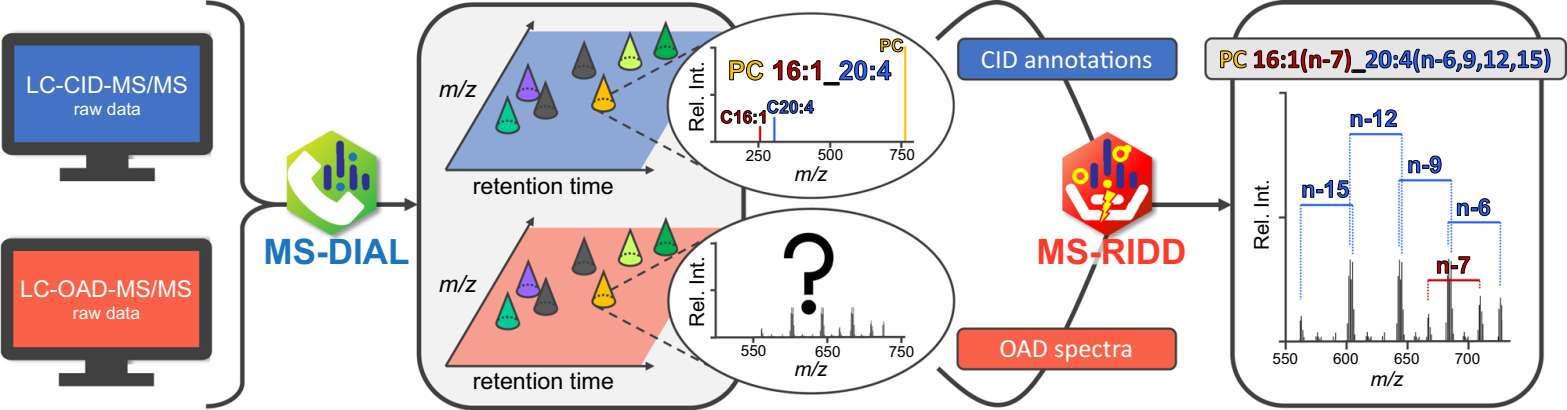
MS-RIDD analysis workflow. Liquid chromatography–mass spectrometry data (LC–MS) are initially processed by MS-DIAL, irrespective of whether they comprise collision-induced dissociation tandem mass spectra (LC-CID-MS/MS) or oxygen attachment dissociation tandem mass spectra (LC-OAD-MS/MS). This step includes peak picking, assignment of MS/MS spectra, and peak alignment. Additionally, based on LC-CID-MS/MS data, MS-DIAL annotates the spectra up to the lipid molecular species level, e.g. PC 16:1_20:4. The results of MS-DIAL are directly utilized by MS-RIDD, which subsequently identifies diagnostic fragment pairs in the LC-OAD-MS/MS data to resolve double bond (C=C) positions. MS-RIDD maps the obtained information to the associated lipid molecular species, providing annotations at the C=C position resolved level, such as PC 16:1(n-7)_20:4(n-6,9,12,15).

Remarkably, Arita et al.^6^ meticulously scrutinized the overall limitations of the composite workflow. They provide comprehensive data on the limit of detection (LOD) and limit of annotation measured based on a total of 79 authentic lipid standards encompassing 23 lipid subclasses since the fragmentation efficiency of C=C generally depends on several variables including the C=C position, lipid subclass, and adduct type. These data may serve as a valuable reference in experimental design.

For an annotation algorithm, the most critical quality criterion is reliability, since it directly relates to the manual efforts required for data curation. A commonly accepted measure for reliability is the positive predictive value (also called precision), which indicates the percentage of correct identifications. However, the question arises of how to acquire ground-truth data with respect to C=C locations in complex lipids. For this purpose, Arita et al.^6^ generated a comprehensive true positive data set comprised of biogenic standards. To generate these standards, lipids were extracted from cells cultured in polyunsaturated fatty acid-enriched media by a well-established procedure^12^. Growth media were supplemented with a set of eight different polyunsaturated fatty acid standards, with single supplements and in various pairings. Compounds detected in the lipid extracts were included in the true positive data set if (i) their abundance increased substantially in the supplemented samples compared to the control and (ii) their MS/MS spectra were of sufficient quality. This allowed for over 200 OAD-MS/MS spectra of more than 50 lipids to be included in the data set to optimize and validate the accuracy of annotations. In the analysis of biogenic standards, MS-RIDD achieved an impressive positive predictive value of 97% in the annotation of C=C positions in OAD spectra. The reliability of assignments was further corroborated by measurements of commercially available standards. As such, MS-RIDD offers precise localization of C=C in a semi-automated workflow, which effectively decreases the required effort in data evaluation.

In order to test the software in a complex biological environment, the robustness and efficacy of the MS-RIDD-based lipidomics platform were demonstrated on a set of seven biological samples, comprised of six mouse samples (brain, eye, skin, liver, testis, and feces) and NIST SRM 1950 human plasma. In total, Arita et al.^6^ reported a remarkable number of 664 unique C=C position-defined structures. A highly interesting outcome of this analysis was the detection of tissue-specific C=C position profiles, which hint at the great potential of this approach to reveal undiscovered lipid pathways and functions.

In summary, Arita et al.^6^ have provided a solid basis for the computer-aided C=C position localization in MS-based lipidomics through a pioneering and expertly designed workflow with appealing annotation quality. Correspondingly, it is easy to imagine that the open-source MS-RIDD software will be extended to other dissociation methods in the future to reach a broader audience. This is of particular interest since other fragmentation techniques are complementary with advantages and limitations in different aspects, such as differing LOD for the individual lipid classes. However, conventional CID features lower LODs for lipid molecular species identification than the LODs that can be obtained for resolving C=C positions using any of the available methods. Accordingly, further developments for both the analytical methods and the computational tools will be pivotal in unraveling the many specific biological functions concealed in the structures of lipids.
